# The Nursing Supplement

**Published:** 1888-09-01

**Authors:** 


### The Nursing Mirror.

All Contributions for this Supplement should be addressed "The Nursing Mirror," care of The Editor, 38, Old Jewry, E.C.

### En Passant.

WHERE WOMEN KILL THEMSELVES.—In reference to the article on "Climate and Suicide," which appeared in our last number, it may interest nurses to know that women kill themselves in greater numbers than elsewhere in the Russian province of Caucasia. In the countries of Europe for every woman there are three or four men who commit suicide; and, as far as is known, the number of men exceeds women in all countries except Caucasia The reason for this exceptional state of matters in Caucasia is not stated.

THE PENSION FUND.—Two cases have lately been brought before the public which show the necessity for a pension fund for nurses. The first is that of Mrs. Duyck, a widow, who supported herself for six years as a nurse, but unfortunately met with an accident some months ago, which has crippled her for life. The second is that of Miss Jessie Baylis, who fractured her heel bone in jumping from a window at the fire in Wigmore Street. Miss Baylis is a Middlesex nurse, and lost all her small possessions; she is being cared for at her *alma mater*, and is doing well, and we hear that the chaplain has collected a nice little sum of money for her.

IT is much wished that existing institutions, such as the admirable institutions at present existing in Glasgow, may offer to affiliate themselves to the Scottish branch of the Queen Victoria Nurses' Institute, under which circumstances they will be required to prove that their nurses reach the required standard, and will conform to the necessary regulations. It is believed that a project is under consideration to give a badge or other decoration to the Queen Victoria nurses. Funds are now required to warrant the Scottish committee in taking and furnishing a suitable training home, as well as subscriptions to supplement the income. Miss Guthrie Wright had the honour of receiving the Queen in one of the sections of the Glasgow Exhibition.

REMEMBER THE NURSES.—It is satisfactory to find that people are beginning to awake to the fact that nurses, like patients and other folks, have "a palate and a set of teeth, and other delicate contrivances to aid digestion," and that work so exhausting to both mind and body as theirs is apt to make their appetite both small and capricious. The following letter, which appeared in "The Lady" of the 2nd ult., contains a sensible suggestion, which we hope will be acted on by many who have more fruit and vegetables in their gardens than they can consume: "Would it be possible for you to make the following suggestions in your paper? For the next few weeks many people living in the country have more fruit and vegetables than they can use. They often send off their superfluity to the hospitals in London and elsewhere. My suggestion is that, instead of sending their hampers merely addressed to the hospital, or 'for the patients,' they should sometimes send them especially 'for the use of the nurses.' The nurses never touch the contents of hampers which are not sent specially for them, and yet they really need the fresh fruit and vegetables more than the patients. The latter are seldom in the hospital longer than one or two months, and have a carefully-arranged and varied diet. The nurses go on month after month with a certain round of joints and puddings, seldom or never varied by fresh fruit or other vegetable than potatoes. Their laborious and self-denying lives claim any little consideration we can show, and I know by experience how grateful they are for thoughtfulness in this particular line.—Believe me, faithfully yours.—A. M. H."

ALL SAINTS' SISTERHOOD. — Over three thousand pounds has already been subscribed towards building a Children's Hospital at Eastbourne as a memorial to Harriett Brownlow Byron, late Mother Superior and Founder of the All Saints' Sisterhood, Margaret Street. The sisters have a beautiful little Convalescent Home at Eastbourne, and their labours there have taught them the need of a hospital also.

THE STRANGE DRUG "FRICTION."—One of the old-fashioned nurses when going her rounds with the doctor one night was ordered to give the patient some friction. The word was strange to her, and having sought in vain for the required substance in the medicine press, she went down to the doctor to ask her if he would give her some friction from the dispensary, as she could not find any in the wards, all the time invoking the reverse of blessings on the day nurses for not leaving it in a blue bottle in the press.

THE ZENANA MEDICAL COLLEGE.—We published a letter from a medical missionary last week, who sent us a report of the above college. We are quite aware of the good work the college does, but would in no way compare the training there received with that received at the School of Medicine for Women. The authorities themselves seem to be aware of their deficiencies, for the report is one long protest against the term "fully qualified." The following details concerning the college may not be uninteresting to many of our readers: The year is divided into three terms, the charge being fifty guineas per annum, which must be paid in advance in full, or in three instalments of £17 10s. each. This charge includes board, residence, and medical instruction. Contributors of one hundred guineas, collected or otherwise, are entitled to nominate a student for two years without further charge, except for clothing, laundry, etc., or contributors of fifty guineas may have the privilege of nominating a student for one year. The institution is open to visitors from three to five p.m. on Mondays and Thursdays. A prayer meeting is held every Saturday from twelve to one o'clock, to which friends are cordially invited.

QUEEN VICTORIA NURSES' INSTITUTE.—Miss Guthrie Wright has written to the Scotch papers about the nursing centre to be established in Edinburgh, with part of the surplus of the Women's Jubilee Fund. The Countess of Rosebery has been appointed president of the Scottish branch and of its provisional committee, of which the first meeting was held on Monday (by the courtesy of the managers) in the board-room of the Royal Infirmary. The following ladies and gentlemen have been nominated: The Marchioness of Lothian, the Countess of Aberdeen, the Hon. Lady Campbell of Blythswood, Lord Hamilton of Dalzell, Mrs. Higgenbotham, and Professor Gairdner, Glasgow; Miss Lumsden, Aberdeen; the Right Hon. J. B. Balfour, M.P.; Dr. Joseph Bell, Sir Thomas Clark, Mrs. Ford, Miss Louisa Stevenson, Mrs. Trayner, and Miss Guthrie Wright. The institute has been named "The Queen Victoria Nurses' Institute." The Queen Victoria nurses will attend the poor in their own houses, under the direction of the local doctors. They will be trained in the Edinburgh Royal Infirmary, and in such other institutions as may meet with the sanction of the committee. They will thus be qualified in efficient hospitals for the work of nurses, including, when necessary, maternity cases after childbirth, and additional training will be given in district nursing. The nurses will be supported by the districts who engage them.

### Original Articles.

### PECULIAR PATIENTS.

Sooner or later, in following their vocation, nurses are sure to come across some specimens of peculiar patients. Every man is liable to illness, and every man needs nursing under those circumstances, so that a nurse who is one day binding the wounds of a burglar in a hospital may the next day be attending to the wants of a sick member of the Royal Family. To a good nurse all men are equal so long as they need her care, and she does not inquire into their character or circumstances, but as convalescence approaches, and cheerful conversation becomes part of the nurse's duty, strange are the stories that are often poured into her ears. The daily papers during one week of the present year contained the following headings with regard to patients who had died in London hospitals: "An Eccentric Drunkard's Death," "Sad Death of a Member of Parliament," "Death of a Female Litigant," "Strange Death of a Governess." All these four persons must have been peculiar patients for the nurses to deal with. The eccentric drunkard was a gentleman of considerable means, who used to retire periodically to the East End to indulge in a drinking bout. Found unconscious outside a common lodging-house, he was taken to the London hospital, and died in a few hours from alcoholism. This story bears some resemblance to the tale of Dr. Jekyll and Mr. Hyde, for the drunkard had two names and two characters—one for his West End home, and the other for his East End haunts. The Member of Parliament was only thirty-six years of age, and was sent to the hospital, because being a bachelor living in chambers he could not there receive the thorough nursing his condition demanded. Unluckily mischief was too far advanced ere he entered the wards, and he grew rapidly worse, and died the same day. The female litigant had been a ladies' maid, and had had some property left her. For thirty years she was an almost constant attendant at the courts and offices, and was always involved in two or three law-suits. She was a quiet, well-behaved woman, and suffered from cancer. Becoming too ill to attend to litigation any longer, she retired into a hospital, and there died. The governess was a German lady, subject to epileptic fits and delusions. Her thigh was fractured in an accident, and not given time to heal. She was found in lodgings, in a very neglected state, and taken to the St. Pancras Workhouse, Infirmary Wing. She was in the prime of life, but quite off her head, and, more than all the others, must have deserved the title of a peculiar patient. Here were four cases which in one week found their way into the public press, but there must have been many more queer patients in the wards that week of whom the outsiders were not cognisant. Perhaps the most pitiful case a nurse can come across is that of the patient who dies unclaimed. An obviously false name is on the entrance ticket, and the dresser extracts but a meagre history from the poor thin lips. The patient may obviously be of a better station than his condition indicates, but the sympathy of the nurse cannot break through the crust of silence in which this man has encased his soul. Probably he is one of those whom a happy family circle speak of with bated breath, "We do not know what became of him, he went to the bad." This is what became of him after a short sinful life—a bed in a hospital ward and the attention of strangers to soothe his dying hours. Even to a nurse inured to sad scenes, the sight of a death-bed where there are no "friends" present causes a shiver; and she cannot help wondering what is the tragic tale which has led to such an end, but which is tightly shut up behind the closed lips which are drawn with pain. Such a patient is more pitiful than peculiar, but others may be strange and amusing. There are the foreigners: the good-natured black sailors, the bright litttle Japanese men, the American with his stock of lies, and the Irishman with his bulls. These, and many others, can be numbered amongst the peculiar patients of whom the public never hears, but whom the nurses know and appreciate.

### THE CRIMEA.

The nurses of the present day who work in well-appointed hospitals have little idea of the labour Miss Nightingale took up when she went to direct the nursing in the Crimea. A few notes of the state of things before her arrival and pending the time she got her system into working order may make us grateful for the blessings we now enjoy, and less likely to grumble at petty troubles. When the cholera first broke out amongst our troops at Varna, the hospital was soon full, and numerous as the medical staff was, and unremitting as they were in their ministrations, there were many painful cases in which the patients were never attended to till too late. Later on a thousand sick and wounded were sent on board the fleet, but still many were left to die of exposure and cold; firewood was scarce, and the watch-fires barely warmed the poor fellows who lay around them wrapped in blankets. In the Russian hospitals gangrene broke out, and great mortality took place. One of the surgeons of a regiment stationed above Balaklava, who had forty sick on his books, was left for three weeks without even the simplest medicines for rheumatism or diarrhœa, from which the men were suffering dreadfully. At one time our army was sending down a thousand sick a day to Scutari, where Miss Nightingale and her band of workers had just arrived. It is not to be wondered at that a hundred of the military doctors fell ill from overwork and disgust. When Sebastopol was at length taken, the *Times* correspondent visited the hospital there, and beheld such horrors as are useless to recall. A long low room, dimly lighted; wounded, dying, dead, and festering corpses all huddled together; the deadly, clammy stench being beyond the endurance of the most experienced surgeons. Many died without a hand to give them a cup of water, or a voice to say one kindly word to them. Then, two hours before an attack was to be made on a redan it was discovered that there was no lint, plaster, or bandages in the camp, and would not be for days. Thankful, indeed, must the soldiers have been to escape from the field hospitals, down to the great red barrack-hospital overlooking the sea at Scutari. Here, after the arrival of Miss Nightingale and her band of fellow-workers, in November, 1854, everything was soon reduced to order; and though there were over two miles occupied by beds only two feet apart, care and attention were bestowed upon all. It is said that the sick used to kiss Miss Nightingale's shadow when it fell on their beds as she passed on her rounds; so great was the chivalrous devotion her tender ministrations aroused. Miss Nightingale not only attended all the death-beds of the soldiers under her charge, but had all the most dangerous cases put in a room next her own, that she might be enabled to render them greater attention. But there was another lady well-known in the Crimea for her attention to the sick and wounded, whose name is now quite forgotten. This was Mrs. Seacole, who had travelled a great deal and was ready for every emergency. She erected an iron house surrounded with wooden sheds, close to Balaklava, and there she took in and tended as many of the sick soldiers as applied to her for relief. During the latter part of the campaign, hers was a familiar name amongst officers and men, and many, though not wounded, delighted to partake of her hospitality. Marvellous tales were told of the cures she effected, but doubtless these cures were chiefly owing to the plentiful stores of nourishment and appliances which she always maintained. Mrs. Seacole was never without lint and bandages and the simpler drugs. She was always in attendance near the battlefield to aid the wounded, and her promptitude, care and cheerfulness saved many a poor fellow's life, and brightened many dreary hours throughout the trying winter of 1855-6, when our troops were wearied by the long campaign and constant hardships. Lucky, indeed, are our nurses in that so few of them know anything of the horrors of war, or have faced experiences so terrible as those met by Mrs. Seacole. Very different is the nursing in an orderly English hospital, with a doctor always at hand, and every possible appliance provided, to the rough-and-ready nursing in the Crimea.

### Letters from a Probationer.—I.

Dear ———, picture me in cap and apron, and already quite at my ease in them. I feel that I have learnt a hundred things during these ten days, and am heartily glad I came. I also see how much there is to learn, and how little it will be possible to achieve in three months. There is a nice garden to the hospital, which we have to cross to the home, and these short breaths of fresh air are simply delicious. I can't tell you how much I miss the fresh country breezes and outdoor life; when I do get an hour off duty, I am too tired to go out; besides, London streets have no attraction to me. The life is much what I pictured it, thanks to the books on nursing you lent me, and the disagreeable sights and sounds one gets used to all too quickly. I was shocked at the callousness of our Sister the first few days, but already my heart is growing stony, and I am glad it is so. We commence work at 7 o'clock, breakfast at 6·30. I have a tiny bedroom to myself with a little bow window. Last night when I pulled up the blind before getting into bed, the moon was visible through the glass-pane on my left hand, this morning, when I was aroused by the bell, there was the same old moon looking complacently down on me, but from the other side. Now I like to sleep "till daylight doth appear," and when I get home won't I revel in long nights of ten hours in bed, instead of a miserable six to seven hours which barely gives you time to go to sleep and wake up again. The air is beautifully fresh before the dawn though—before the smoke from millions of chimneys has clogged it with soot—and the outlines of this huge building stand out clear and distinct in the faint grey light. Perhaps you don't know that Venus is a morning star just now, but I do. When I enter the ward in the morning, all the patients say "Good-morning" as I pass them, and I reply, but this morning I simply nodded to one small boy, upon which he shouted after me, "Why don't you say 'Good-morning' Nuss? Where's your manners?" They are a good-humoured respectful lot of men I wait on, but I wish there weren't so many cases complicated by D. T. To be asked to remove snakes, and to hear occasional outcries of "murder!" 'is rather trying to the nerves. My dear friend, *could* you send me something nice to eat? It sounds very dreadful doesn't it, after all the high-falutin nonsense I talked about a life of self-denial, but I am losing my appetite, and the food here may be wholesome, but is not enticing. Such thick bread and butter as we were never given even in the nursery at home! And though I daresay I shall relish it when I get used to it, I might make the descent more easily and comfortably if you could send me such a box as a school-boy loves. Do you remember a tall woman we used to speak to at Mentone last year? We called her "Velvet" between ourselves; well, she is the sister of one of the wards here and is such an awful martinet. The nurses call her ward "purgatory," and declare that matron sends you there as punishment if you offend elsewhere. She never will apply for a special nurse, no matter how many bad cases she has, and I believe she scolds by the hour. I dread being sent there; just fancy a woman of my age (I am sure I am as old as she is) standing up to be scolded! Such funny memories of the old school days come back to me when we go trooping across to dinner in flocks, and when I have to tidy my own room. There are lectures too by some of the doctors, and we all sit in rows on long forms, while he chalks up things on the blackboard and bids us take notes. Yet how different is the work, and how great the responsibilities in this life upon the threshold of which I stand!

### Appointments.

[It is requested that successful candidates will send a copy of their applications and testimonials, with date of election, to The Editor, The Lodge, Porchester Square, W.

Miss Lydia Constable, who has been for the last seven years a sister at St. Marylebone Infirmary, Notting Hill, and who received her training in the Nightingale School, St. Thomas's Hospital, has been appointed matron at the Gordon Boys' Home, Chobham, near Woking.

Miss Rhoda Smith, now at the Bury Infirmary, has been appointed matron of the Victoria Hospital, Swindon. There were 71 applicants. Miss Smith received her training in medical and surgical work at the Bristol Royal Infirmary, and has had over nine years' experience. The hospital, which has been built in commemoration of the Queen's Jubilee, and which her Majesty has commanded shall be called The Victoria Hospital, will be opened for the reception of patients on Saturday, September 29th next.

Warneford Hospital, Leamington.—Miss Price has been appointed matron of this hospital. Miss Price was trained at Addenbrookes, and several times took matron's duty while she was there, and thus secured knowledge of housekeeping, etc. Afterwards Miss Price went to Dulwich Infirmary, where she had charge of the children's and other wards. The Addenbrookes nurses have secured several good appointments lately, and are to be congratulated. Miss Oldham, whose appointment to Shrewsbury we chronicled some time since, also got her first three months' training as a paying probationer at Addenbrookes.

### One Day at a Time.

By Helen Hunt Jackson.

One day at a time! That's all it can be;
No faster than that is the hardest fate;
And days have their limits, however we
Begin them too early and stretch them too late.

One day at a time!
It's a wholesome rhyme!
A good one to live by,
A day at a time.

One day at a time! Every heart that aches,
Knowing only too well how long they can seem;
But it's never to-day which the spirit breaks—
Its the darkened future, without a gleam.

One day at a time! But a single day,
Whatever its load, whatever its length;
And there's a bit of precious scripture to say
That, according to each, shall be our strength.

One day at a time! 'Tis the whole of life;
All sorrow, all joy, are measured therein:
The bound of our purpose, our noblest strife,
The one only countersign sure to win!

One day at a time!
It's a wholesome rhyme!
A good one to live by,
A day at a time.

### Everybody's Opinion.

[*Correspondence on all subjects is invited. No communications can be entertained if the name and address of the correspondent is not given, or unless one side of the paper only be written on.*]

### NURSING AT REDUCED RATES.

Some weeks ago I sent you, as requested in The Hospital, the new report of the Nurses' Home, in Bethel Street, Norwich, of which I am the superintendent. I have noticed the correspondence on the subject of nursing at reduced rates—those whose incomes prevent their paying the higher fee, and are yet in need of skilled nursing. This has been done for the past twenty-one years by the Norfolk and Norwich Staff of Nurses, in addition to gratuitous nursing of the poor in their homes—not district work—on the certificate of a medical man. There are thirty nurses now on the staff—medical, surgical, and monthly—who of course are frequently engaged at full fee. Subscriptions and donations also are gladly taken to enable us to undertake the more charitable work—the work which the committee have most at heart.

Edith Watson,
Lady Superintendent of Nurses' Home, Norwich.

### FREE TRAINING IN MIDWIFERY.

I have been told that free training in midwifery is given at Brownlow Hill, Liverpool. I cannot speak for the truth of it, but it might be useful to some of your numerous readers to know. The training given at the City Road Lying-In Hospital is much less expensive than Queen Charlotte's.

One of the Many Admirers of "The Hospital."

### THE TITLE OF NURSE.

May I beg leave to add a few remarks to letter of the 18th of August, signed "Another Old Nurse?" "An Old Nurse" seems to be anything but proud of being called nurse. Could she have been in my position for the last ten months, I think "An Old Nurse" would have been glad to have heard the name addressed to her sometimes. Last October I undertook a post to a lady as nurse, maid, and masseuse. I had been private nursing, and thought a permanent case would give me more rest at night for a time, never dreaming for one minute that I should be supposed to drop my title to the name of nurse. I wrote, offering my services, saying I knew nothing about maid's work, but was a trained nurse, medical rubber, &c., and as my patient was partly paralysed, of course I had to nurse her; but I had not been in the house long, before I found myself addressed, instead of nurse, as Jones—thus: Jones that, and Jones do this, and when addressed as "nurse" by the servants of the house I was told I was not to allow it. When I remarked I could not tell them not to, as I was only too proud to be called nurse, I was told, "Well, you will not get them to pay you the respect you would if you were treated as my maid." I only know that I can heartily say I never have had a more miserable ten months than I have had as nurse and maid, and whilst being treated as a maid. I have been a private nurse for more than eleven years now, and, with a very few exceptions, in all the houses I have been in I have been treated with the greatest kindness and consideration; in fact, at times almost spoilt. Nothing has been thought too good for nurse, and I am happy to say I intend shortly to join an institution again; and although I only get about half the salary I should get if I remained here, I prefer to be called nurse and be happy in my work; and I am sure the oftener I hear "nurse" instead of "Jones" the better I shall like it.

Another Old Nurse.

### Notes and Queries.

To Correspondents.—1. Questions may be written on post-cards. 2. Advertisements in disguise are inadmissible. 3. In answering a query please quote the number. 4. If a private answer is desired, a stamped addressed envelope must be forwarded.

(47) Will you kindly tell me the price of Bell's "Surgery for Nurses," and by whom it is published. Madge. The price is 2s. 6d., and the book is published by Oliver and Boyd, Edinburgh.

(48) *Assistant Nurses.*—Will you please tell me the names of the hospitals where they take assistant nurses?—Holiday Nurse.

(49) A. N. writes: Will you be so kind as to let me know how I could get to Nice, in France, as a private nurse? I wish to join the Private Nursing Establishment at Nice, and should be so glad if you could let me know about this in your next week's paper.

**Answers.**

(44) Nurse A. can buy the turn-down Squire collars 4s. 6d. per dozen. and also the Deep Eton collars, same price, or a little deeper than the usual Eton collars, specially made to order for 5s. 6d. per dozen from Messrs. Robinson and Cleaver, Belfast, Ireland.

Messrs. Wallace and Co., Holborn Circus, London, supply bonnets and cloaks, the same as worn by Bart's nurses. Nurse Thomas.

### Vacancies.

The following vacancies are announced:—

**Matrons.**

Bedford General Infirmary; £50.

Great Yarmouth New Hospital; £35.

Jamaica Lunatic Asylum; £160.

Harrogate Cottage Hospital; £40.

**Lady Superintendents.**

West Kent General Hospital; £60.
Leicester Institution of Trained Nurses; £40.

**Nurses.**

Windsor Infirmary; £20.

Great Yarmouth New Hospital; £22.

Dispensary Hospital, Bury; £25.

Kent Nursing Institution.

Albert Hospital, Devonport.

Ryde Institute.

Brighton Borough Fever Hospital; £25.

Homes for Aged Mariners, Egremont, Cheshire; £25.

C. C. Poorhouse, Irvine, Scotland; £25.

Brighton and Hove Hospital for Women; £30.

Mrs. Mann's Surgical Home; £22.

Leeds Fever Hospital; £25.

Canterbury Institute.

Swansea Nursing Institution.

Victoria Home, Bournemouth.

Miss John's Home, Glasgow.

Bradford Eye and Ear Hospital; £25.

Radcliffe Infirmary, Oxford; two.

Nurses' Home and Training Institution, Sheffield; several.

Kent Nursing Institution; several.

Southport Trained Nurses' Home.

Bristol Royal Infirmary; £25 to £28.

**District Nurses.**

Madeley. Stamford. Southsea.

**Probationers.**

Workhouse Infirmary Nursing Association.

Nurses' Institute, Canterbury.

Royal Hospital for Diseases of the Chest.

### Exchange and Mart.

Under this heading we propose to insert purely Exchange advertisements, at a charge of sixpence for eighteen words, or three insertions for one shilling It must be distinctly understood that ordinary advertisements will not be inserted here.

WANTED.—"Quain's Dictionary," in fair condition. Would give a pocket spectroscope, which cost twenty-five shillings. Address Student, Office of The Hospital.

APRONS.—Half a-dozen white linen aprons with bibs. Will exchange for nurse's surgical case. Nurse Norma, Office of The Hospital, 38, Old Jewry, E.C.

### DISCIPLINE FOR THE INSANE.

In an asylum, discipline or moral suasion—call it what you will—ought to be applied as far as possible to the individual; and there, as in the school class, the more it is distributed, the less powerful its effects. You will perhaps be told that when a person becomes insane his or her individuality becomes changed; that may be so, but all the more must that changed individuality be submitted to particular and individual moral influences.

A Missing Hospital Nurse.—J. J. A. writes: "E. M. W. the probationer who left the Chest Hospital without permission, on Bank Holiday, August 6th, is now safely at home with her family. She met with an accident which necessitated her admission to the hospital of a provincial city, whence as soon as she was able she wrote to her father, who lost no time in having her removed to her home where she is now convalescent."

